# CCL25 chemokine promotes antiviral tissue resident CD4^+^ and CD8^+^ effector memory T_RM_ cells associated with a reduction of ocular herpes infection and disease: a potential gut–eye axis in herpes immunity

**DOI:** 10.3389/fimmu.2026.1872553

**Published:** 2026-07-17

**Authors:** Azizur Rahman, Swayam Prakash, Sweta Karan, Sarah Xue Le Ng, Gina Park, Chhaya Maurya, America Garcia, Khan Intharachalit, Junseong Hwang, Celine Tze Yao Tang, Reilly Andrew Chow, Baverly Sabathini Suoth, Emma Jane Liao, Lbachir BenMohamed

**Affiliations:** 1 Laboratory of Cellular and Molecular Immunology, Gavin Herbert Eye Institute, School of Medicine, University of California, Irvine, Irvine, CA, United States; 2 Institute for Immunology, School of Medicine, University of California, Irvine, Irvine, CA, United States; 3 Department of Vaccines and Immunotherapies, TechImmune, Inc., University Lab Partners, Irvine, CA, United States

**Keywords:** CCL25, CD4+ and CD8+ T cells, HSV-1, mucosa, ocular herpes

## Abstract

CCL25, a mucosal chemokine mainly produced in the gut, is known for its role in intestinal immunity through CCR9. In this study, we tested whether CCL25 also contributes to ocular immunity within the gut-eye axis during herpes simplex virus type 1 (HSV-1) infection. Using CCL25-deficient (CCL25^(–/–)^ deficient) mice and wild-type C57BL/6J controls, which were infected ocularly with HSV-1 (McKrae strain), we found that CCL25 deficiency led to more severe corneal disease, increased viral replication, and reduced survival. CCL25^(–/–)^ deficient mice also had fewer antiviral effector memory CD44^+^CD4^+^ and CD44^+^CD8^+^ T cells in the cornea, gastrointestinal tract, and draining lymph nodes, as well as reduced numbers of activated tissue-resident CD69^+^CD103^+^CD4^+^ and CD69^+^CD103^+^CD8^+^ T_RM_ cells that produced interferon-γ. Immunohistochemistry showed strong CCL25 expression in the gut of wild-type mice but not in the cornea or conjunctiva. In addition, the mucosal chemokines CXCL14, CXCL17, and CCL28 were upregulated in infected ocular tissues of wild-type mice but remained low in CCL25^(–/–)^ deficient mice, suggesting that CCL25 influences the expression of major mucosal chemokines. Collectively, these findings indicate that CCL25 supports recruitment of antiviral corneal tissue-resident effector memory T_RM_ cells during ocular HSV-1 infection and supports the concept of a gut-eye axis in herpes immunity. The CCL25/CCR9 pathway may represent a promising therapeutic target to protect from herpetic eye disease.

## Introduction

Herpes simplex virus type 1 (HSV-1) is the leading cause of infectious corneal blindness in the developed world, with an estimated 1.5 million cases of HSV keratitis annually and approximately 40,000 new cases of severe visual impairment or blindness each year (1). The most vision-threatening manifestation is recurrent herpetic stromal keratitis (HSK), a chronic immunoinflammatory condition driven by repeated HSV-1 reactivation in the corneal stroma, leading to progressive immunopathology, corneal scarring, neovascularization, and permanent vision loss (2–5). Effective control of HSV-1 and prevention of recurrent HSK depend on the timely recruitment of virus-specific effector T cells into the infected cornea (6, 7). Both CD4^+^ and CD8^+^ T cells are critical mediators of corneal anti-HSV-1 immunity: CD4^+^ T cells are primed in the draining lymph nodes and restimulated within the infected cornea to regulate the destructive inflammatory disease characteristic of HSK (8–12). In contrast, CD8^+^ T cells provide direct antiviral control through IFN-γ secretion and cytolytic mechanisms (13–15). Delayed or insufficient recruitment of these effector T cell populations permits unrestrained viral replication, fueling recurrent inflammatory cascades that define HSK and ultimately threaten vision (7).

Chemokines are chemotactic cytokines that govern the migratory patterns and positioning of all immune cells, orchestrating both innate and adaptive immune responses during infection, inflammation, and tissue homeostasis (16–18). Among the 48 known chemokines, four — CCL25, CCL28, CXCL14, and CXCL17 — are especially important in mucosal immunity because they are homeostatically expressed in mucosal tissues and play critical roles in directing T cell trafficking and immune surveillance at mucosal surfaces (17, 19–21). CCL25 is primarily expressed by epithelial cells of the small intestine and thymus, where it recruits CCR9-expressing T cell subsets; CCL28 is expressed broadly across mucosal epithelia and recruits CCR10^+^ effector T cells and IgA-secreting plasma cells; while CXCL14 and CXCL17 contribute to mucosal macrophage recruitment and direct antimicrobial defense (19, 22). The identification of these epithelial-expressed mucosal chemokines underscores their pivotal role in directing T cell localization at mucosal surfaces under both homeostatic and inflammatory conditions, enabling the selective accumulation of distinct tissue-resident memory (T_RM)_ cell subsets within mucosal tissues (17, 23). In the context of herpes simplex virus (HSV) infection, the four major mucosal-associated chemokines CCL25, CCL28, CXCL14, and CXCL17 play an important role in protecting mucosal surfaces from incoming infectious pathogens by mobilizing antiviral effector memory T cells and memory B cells to sites of herpes infection (24). Importantly, corneal, and conjunctival epithelial cells have been identified as potential sources of the mucosal chemokines CCL28, CXCL14, and CXCL17 at the ocular surface, with their expression upregulated under inflammatory conditions (25). However, the role of the gut-derived mucosal chemokine CCL25 and its receptor CCR9 in regulating ocular HSV-1 infection and corneal T cell immunity remains entirely unexplored.

The CCL25 gut mucosal chemokine (also known as thymus-expressed chemokine, or TECK) directs T cell migration to the gut *via* its receptor, CCR9 (22, 26). CCL25 “imprints” T cells with a CCR9-positive gut-homing profile in the gut. In the thymus, TECK recruits T cell precursors during their development (27). Although the role of CCL25 in gut immunity is well understood, its influence on ocular infection and immunity remains largely unexplored. Since ocular tissues do not produce CCL25, any effect on immune control of the eye would be secondary to systemic immune modulation rather than to local chemokine gradients (27). Effector memory T cells are crucial for immediate immune defense in peripheral tissues, as they function without reactivation in lymphoid organs (28, 29). Overlapping chemokine systems can facilitate their recruitment to non-gut sites. This suggests that CCL25 may indirectly influence ocular immunity by shaping chemokine networks or by maintaining a systemic reservoir of ready-to-deploy effector memory T cells.

In this study, to test this hypothesis, and in light of the current emerging concept of “gut-eye immune axis”, a dynamic, bidirectional immune network between the gut and eye, we investigated the role of CCL25 chemokine in ocular herpes infection and immunity using corneal infection with simplex virus type 1 (HSV-1) as a model. We evaluated the severity of viral replication in eye swabs, corneal disease, and survival. We correlated these with the frequency and function of local antiviral IFN-γ- producing cytotoxic T cells infiltrating the cornea, trigeminal ganglia (TG), and draining lymph nodes, as well as with mucosal chemokine expression profiles. Our findings support the concept of the “gut-eye immune axis” and shed light on how the gut-derived CCL25 chemokine promotes the migration of protective effector CD4^+^ and CD8^+^ T cells to the cornea and conjunctival mucosal tissue of the eye, as well as to non-mucosal sites, thereby enhancing ocular herpes immunity. The finding suggests the potential for targeting CCL25 pathways in ocular immunotherapy to enhance protection against ocular herpes.

## Materials and methods

### Mice

Female CCL25 knockout (CCL25^(–/–)^) mice on the C57BL/6 background, 6–8 weeks old, were purchased from Taconic Biosciences (Germantown, NY). Female C57BL/6 (B6) mice (6–8 weeks old) were purchased from the Jackson Laboratory (Bar Harbor, ME). The mice were maintained and bred under specific pathogen-free conditions with a 12-hour light/dark cycle and access to food and water (*ad libitum*). Animal studies conformed to the Guide for the Care and Use of Laboratory Animals published by the U.S. National Institute of Health. All procedures were approved by the Institutional Animal Care and Use Committee (IACUC) of the University of California, Irvine (Protocol # AUP-22-086) and conducted in accordance with the NIH guidelines.

### Virus propagation and titration

HSV-1 strain McKrae, a virulent ocular isolate, was propagated in confluent rabbit skin (RS) cell monolayers (ATCC, Manassas, VA) cultured in Minimum Essential Medium (MEM) supplemented with 10% fetal bovine serum (FBS) and 1% penicillin-streptomycin. Once an extensive cytopathic effect was observed, infected cultures were harvested by freeze–thaw lysis. Viral titers, expressed as plaque-forming units (PFU), were determined by standard plaque assay on RS cells.

### Ocular HSV-1 infection mode

Female C57BL/6 wild-type (WT) and CCL25^(–/–)^ deficient mice (6–8 weeks old) were anesthetized with isoflurane. The central corneal epithelium of each eye was lightly scarified with 6–8 superficial crosshatch scratches using a sterile 30-gauge needle. Immediately afterward, 2.5×10^5 PFU of HSV-1 in 5 µL phosphate-buffered saline (PBS) was applied to the corneal surface, ensuring uniform coverage. Mice were monitored until they recovered from anesthesia, after which they were returned to their cages.

### Clinical scoring of ocular disease

**Figure 1:**
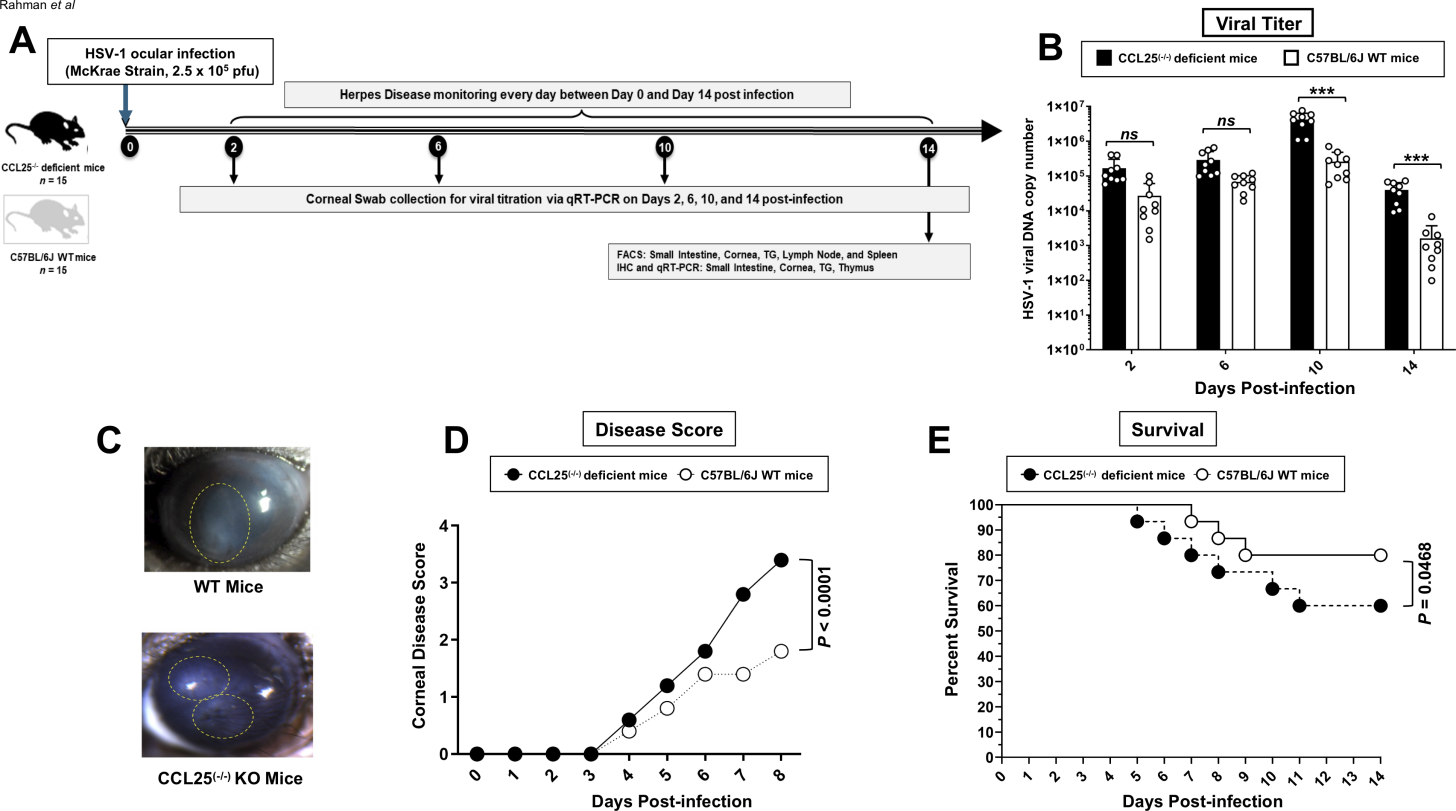
CCL25 deficiency worsens HSV-1–induced corneal disease, increases viral load, and reduces survival. **(A)** Schematic of experimental design. Wild-type (WT) and CCL25^(–/–)^ deficient mice were ocularly infected with HSV-1 and monitored daily for corneal disease from day 0 to day 14 post-infection. Corneal swabs were collected on days 2, 6, 10, and 14 to quantify viral load by qRT-PCR. Tissues, including the small intestine, cornea, trigeminal ganglia (TG), and lymph nodes, were analyzed by flow cytometry, while immunohistochemistry (IHC) was used to assess chemokine expression. **(B)** Viral load in corneal swabs, measured by qRT-PCR. CCL25^(–/–)^ deficient mice exhibited significantly higher HSV-1 DNA copy numbers on days 2, 6, and 10, reflecting impaired viral clearance compared to WT mice. **(C)** Representative corneal images of WT and CCL25^(–/–)^ deficient mice following HSV-1 infection. WT mice showed only mild, transient haze that resolved by day 14, whereas CCL25^(–/–)^ deficient mice developed earlier and more severe corneal opacity with neovascularization, which persisted through day 14. **(D)** Corneal disease scores over time. WT mice displayed significantly milder disease compared to CCL25^(−/−)^ deficient mice between days 4 and 8 post-infection (two-way ANOVA, ***P ≤ 0.001). **(E)** Kaplan–Meier survival analysis. WT mice showed higher survival (80%) than CCL25^(–/–)^ deficient mice (60%) during the 14-day infection period (log-rank test, *P* = 0.0468).

Following infection, mice were examined daily for 14 days for signs of ocular herpes disease. Key clinical signs monitored included corneal opacity (haze), corneal neovascularization, blepharitis (eyelid inflammation), conjunctival redness, and any neurological symptoms (e.g., head tilt or impaired coordination) indicating severe infection spread (**Figure 1A**). Disease severity in each eye was scored on a 0–5 scale based on corneal involvement: 0 = clear, normal cornea with no disease; 1 = mild superficial keratitis or eyelid redness; 2 = moderate stromal inflammation with mild corneal haze (iris detail visible); 3 = severe corneal opacity obscuring the iris; 4 = dense corneal opacity with extensive neovascularization or ulceration; and 5 = corneal perforation or end-stage disease (often accompanied by severe neuroinvasive signs or phthisis bulbi). Trained observers, blinded to mouse genotype, performed the scoring. Representative ocular photographs were obtained at multiple time points using a slit-lamp biomicroscope or a stereomicroscope equipped with a digital camera to document disease progression.

### Flow cytometry

To characterize immune cell responses, we prepared single-cell suspensions from multiple tissues of HSV-1–infected mice at the peak of disease (days 7–8 post-infection). Corneas, trigeminal ganglia (TG), cervical draining lymph nodes (CLN), spleen, and small intestine segments were harvested and enzymatically digested with collagenase D (15 mg/mL in RPMI 1640) for 1 hour at 37 °C to release leukocytes. Dissociated cells were passed through a 70 µm strainer and washed with RPMI containing 2% FBS. Single-cell suspensions were first gated on scatter parameters (FSC-A vs. SSC-A) to exclude debris, followed by doublet discrimination (FSC-H vs. FSC-A), and viability gating using a live/dead fixable viability dye to exclude non-viable cells before immunophenotyping. Lymphocytes were subsequently gated on CD45^+^ cells to confirm leukocyte identity before T-cell subset analysis. Compensation controls consisted of single-stain preparations using antibody capture beads for each fluorochrome, and fluorescence minus one (FMO) controls were employed for all markers used to define activation and tissue-residency status, including CD44, CD69, and CD103, to ensure accurate gate placement in the presence of background fluorescence. The gating strategy is provided in **Supplementary Figure 1**.

For surface marker staining, ~10^6 cells per sample were resuspended in FACS buffer (PBS, 1% FBS, 0.1% sodium azide) and incubated for 45 minutes at 4 °C with fluorochrome-conjugated monoclonal antibodies against CD45, CD3, CD4, CD8, CD44, CD62L, CD69, CD103, and B220 (all from BD Biosciences). Following surface staining, cells were fixed and permeabilized using BD Cytofix/Cytoperm according to the manufacturer’s protocol. Intracellular IFN-γ staining was performed in Perm/Wash buffer on ice for 30–45 minutes with anti-mouse IFN-γ. Samples were acquired on a BD flow cytometer, and data were analyzed with FlowJo software. Lymphocyte populations were identified by forward- and side-scatter gating and CD45^+^ gating. T cells were defined as CD3^+^, with further subdivision into CD4^+^ and CD8^+^ subsets. Effector-memory T cells were classified as CD44^+^ CD62L-, activation status was determined by CD69 expression, and tissue-resident memory T cells by CD103 expression. Intracellular IFN-γ^+^ cells represented virus-specific cytokine-producing T cells.

### Immunohistochemistry of chemokine detection and histopathology

Eyes, trigeminal ganglia, and small-intestine samples were collected, embedded in OCT, flash-frozen, and cryosectioned (10µm) onto Superfrost Plus slides. Sections were air-dried, fixed in 4% paraformaldehyde, rinsed, and blocked/permeabilized in PBS with 5% normal donkey serum and 0.3% Triton X-100. Primary antibodies against CCL25, CCL28, CXCL14, and CXCL17 were applied overnight at 4 °C, followed by Alexa Fluor–conjugated secondary antibodies for 1 hour in the dark. Nuclei were counterstained with DAPI, and slides were mounted with Fluoromount-G. Images were captured on a Keyence BZ-X710 fluorescence microscope with identical exposure settings for WT and CCL25^(–/–)^ tissues, and fluorescence intensity/co-localization was quantified using ImageJ (Version 1.54p).

For hematoxylin and eosin (H&E) staining, the mouse cornea and TG sections were fixed in 4% PFA for 48 hours, then transferred to 70% ethanol. The tissue sections were then embedded in paraffin blocks and sectioned at 8 μm. Slides were deparaffinized and rehydrated before being stained with hematoxylin and eosin (H&E) for immunopathological analysis. Images were captured using the BZ-X710 all-in-one fluorescence microscope (Keyence). The histopathological changes were measured by ImageJ (Version 1.54p), to quantify the percentage area of infiltrated satellite cells in the Cornea and TG of CCL25^-/-^ mice and WT mice’s H&E-stained cornea and TG sections.

Inflammatory infiltrates were quantified by counting hematoxylin and eosin-stained sections using a standardized scoring system, with a minimum of five non-overlapping fields per section analyzed at consistent magnification across all samples. A minimum of three to five corneal or TG sections per animal were evaluated, with group sizes consistent with those reported in the corresponding flow cytometry and viral burden analyses. Critically, all histopathological scoring was performed by an investigator blinded to animal genotype to eliminate observer bias. Satellite cells, which refer specifically to the FABP7^+^ satellite glial cells that closely surround neuronal cell bodies within the TG, are morphologically distinguishable from infiltrating immune cells and neurons in H&E-stained sections.

### Virus titration through quantitative real-time PCR

Corneal swabs were collected from both eyes using a Dacron swab (type 1) (Spectrum Laboratories, CA, USA) on Days 2, 6, 10, and 14 post HSV-1 McKrae infection. Individual swabs were transferred to a 2 mL sterile cryogenic vial containing 1 mL culture medium and stored at -80 °C until use. HSV-1 viral DNA was isolated from these corneal swab samples using a Quick-DNA™ Viral Kit (Zymo Research, USA). HSV-1 titers in tear samples were determined by real-time quantitative PCR, as previously described (30, 31).

Viral titers in corneal swabs were quantified by qPCR targeting the HSV-1 glycoprotein B (gB) gene and normalized to host genomic DNA using the mouse Actb gene as a reference, ensuring that differences in sample cellularity or swab efficiency did not confound viral load comparisons between groups. The qPCR-based measurements reflect total viral genome copies rather than infectious virus per se. To validate these findings and confirm the presence of replication-competent virus, corneal homogenates from a subset of animals were subjected to standard plaque assay on Vero cell monolayers, and the results were concordant with the qPCR data.

### Statistical analysis

Data for each assay were compared by ANOVA and Student’s *t*-test using GraphPad Prism version 10.5.0 (La Jolla, CA). The physical estimation data were analyzed using a paired t-test, based on a nonparametric Gaussian distribution, as determined by the Wilcoxon matched-pairs signed-rank test. Data are expressed as the mean ± SD. Results were considered statistically significant at a *P* < 0.05.

## Results

### 1 CCL25 deficiency leads to severe ocular herpetic disease, higher viral loads, and death following ocular HSV-1 infection

To investigate the direct role of CCL25 in corneal herpes simplex virus type 1 (HSV-1) infection and immunity, we generated CCL25^(–/–)^ deficient mice, which are deficient in CCL25, and compared their ocular herpes infection, disease and death with those in age-matched wild-type (WT) C57BL/6J littermates (*n* = 15 mice per group) following an ocular inoculation with HSV-1 (2.5 x 10 (5) McKrae strain) (**Figure 1A**). Compared to WT controls, we found that on days 8 and 14 post-infection the corneas of CCL25^(–/–)^ deficient mice carried relatively higher HSV-1 DNA copy numbers at days 8 and 14 compared to the corneas of wild-type (WT) C57BL/6J mice (**Figure 1B**). Similarly, compared to WT controls, we found that on day 2 following ocular HSV-1 infection, the corneas of CCL25^(–/–)^ deficient mice looked similar to those of wild-type (WT) controls. However, by day 8, apparent differences emerged: CCL25^(–/–)^ deficient mice developed pronounced corneal opacity and extensive neovascularization, which persisted through day 14, whereas WT corneas showed only mild haze that gradually resolved (**Figure 1C**). These observations were supported by disease scoring (**Figure 1D**). In CCL25^(–/–)^ deficient mice, disease scores climbed rapidly, reached higher peaks, and remained elevated for longer. In contrast, WT mice exhibited a more moderate course, which stabilized and improved by the end of the observation period. Statistical analysis confirmed that disease severity was significantly greater in the knockout group (two-way ANOVA, ****P < 0.0001). The increased pathology in CCL25^(–/–)^ deficient mice was also reflected in the survival outcomes (**Figure 1E****).** By day 14, survival was reduced to ~60% in CCL25^(–/–)^ deficient mice compared to ~80% in WT mice, a significant difference (log-rank test, *P = 0.0468). Viral clearance mirrored these clinical outcomes.

Collectively, these data highlight a central role for CCL25 in limiting HSV-1 corneal disease. In its absence, uncontrolled viral replication and heightened inflammation drive more severe keratitis and increased mortality, likely due to impaired recruitment or function of protective immune cells. These findings underscore the broader importance of CCL25-mediated mucosal immunity in antiviral defense and suggest that the CCL25 axis may be a therapeutic target to enhance host resistance while minimizing corneal damage.

### 2 CCL25 is required for corneal CD69^+^CD103^+^ tissue-resident memory CD4^+^ and CD8^+^ T cell establishment and activation during HSV-1 infection

**Figure 2:**
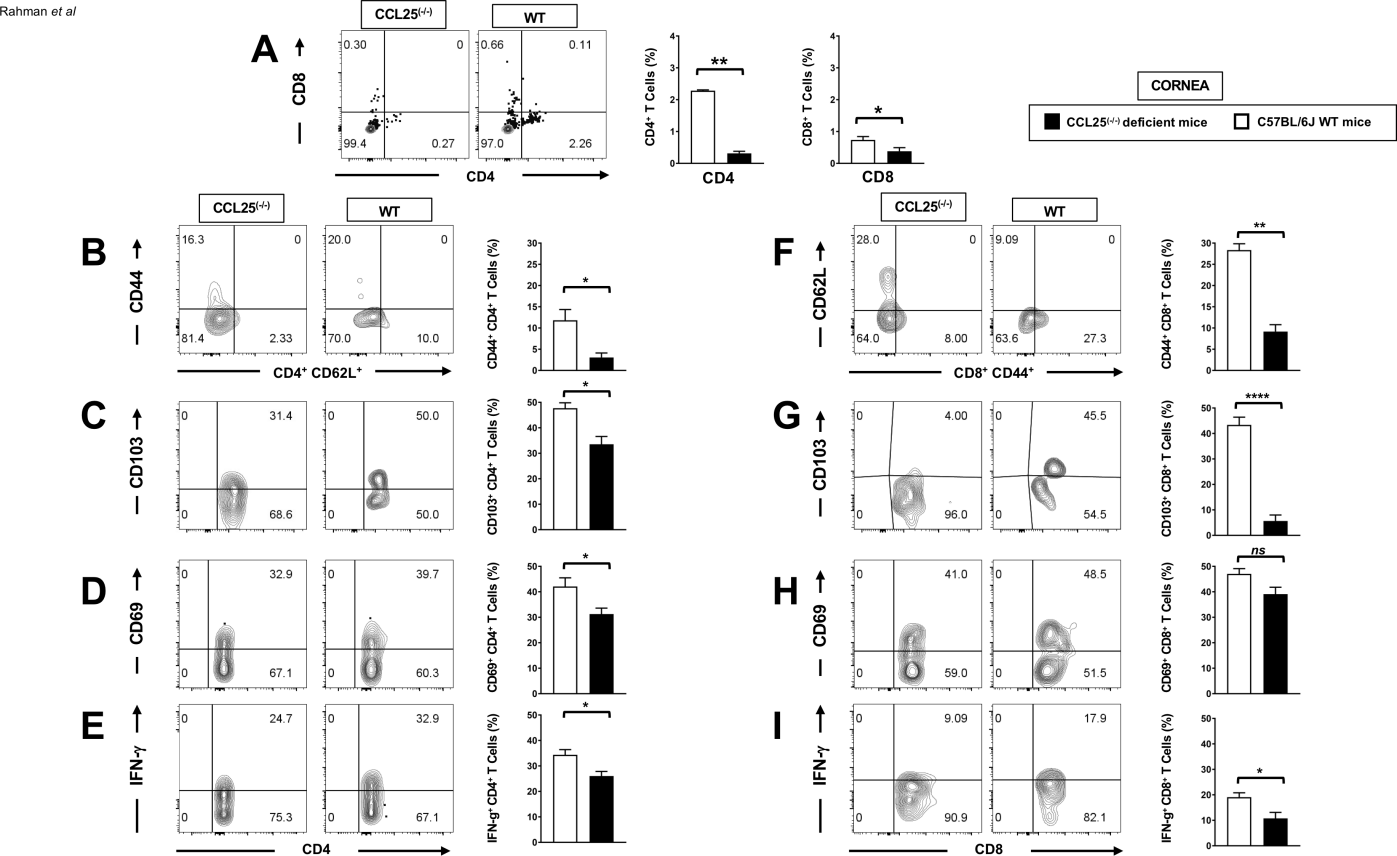
Corneal T-cell responses (CD4^+^/CD8^+^) and residency/activation markers depend on CCL25 during HSV-1 **(A)**. Frequencies of CD4^+^ and CD8^+^ T cells were higher in WT corneas than in CCL25^(–/–)^ deficient mice. **(B)**. WT CD4^+^ T cells showed more effector-memory cells (CD44+T), while CCL25^(–/–)^ deficient mice had fewer effector memory cells (CD44+T). **(C, D)** WT CD4^+^ T cells expressed significantly higher levels of T_RM_ markers CD103 and CD69. **(E)** WT CD4^+^ T cells produced more IFN-γ, reflecting stronger antiviral function. **(F)** WT CD8^+^ T cells were enriched for effector-memory subsets, unlike CD4 T cells. **(G, H)** WT CD8^+^ T cells also expressed higher CD103 and CD69, consistent with T_RM_ cell differentiation, whereas these subsets were reduced in CCL25^(–/–)^ deficient mice. **(I)** IFN-γ production by CD8^+^ T cells was significantly greater in WT mice than in CCL25^(−/−)^ deficient mice. Together, these data show that CCL25 is required for the development and function of corneal CD4^+^ and CD8^+^ T cells during HSV-1 infection. *P ≤ 0.05, **P ≤ 0.01, ****P ≤ 0.0001.

Flow cytometry of corneal infiltrates showed apparent differences in T cell responses between WT and CCL25^(–/–)^ deficient mice during HSV-1 infection. In the corneas of WT mice, both CD4^+^ and CD8^+^ T cells were present at higher frequencies compared to CCL25^(–/–)^, with CD4^+^ T cells showing the most significant increase (**Figure 2A****).** Phenotypical analysis revealed a reduced frequency in CCL25^(–/–)^ deficient mice for CD44^+^ T cells. In contrast, WT mice had a greater proportion of CD44^+^ effector-memory T cells in both CD4^+^ and CD8^+^ compartments (**Figures 2B, F**), indicating a more substantial shift toward effector differentiation.

Markers of tissue residency also highlighted a genotype-dependent difference. Among CD4^+^ T cell subsets, CD103 and CD69 expression were relatively higher in WT mice than in CCL25^(–/–)^ deficient mice (**Figures 2C, D**). The CD103 and CD69 expression was higher in WT mice compared to CCL25^(–/–)^ deficient mice, particularly among CD8^+^ subsets, suggesting enhanced generation or retention of corneal T_RM_ cells (**Figures 2G, H****).** Despite these increases in effector-memory and T_RM_ populations, functional analysis showed that fewer CCL25^(–/–)^ T cells produced IFN-γ than WT cells in both the CD4^+^ and CD8^+^ compartments (**Figures 2E, I**).

Together, these findings demonstrate that CCL25 is indispensable for the development of robust mucosal T cell immunity during HSV-1 infection. In WT mice, CCL25 promotes the recruitment and retention of CD4^+^ and CD8^+^ T cells with a T_RM_ cell and effector memory phenotype (CD44^+^ T cells, CD103^+^ T cells, CD69^+^ T cells). It enhances their effector function through increased IFN-γ production. In contrast, CCL25^(–/–)^ deficient mice fail to generate strong T_RM_ cells and effector memory phenotype responses and exhibit impaired cytokine production, which likely contributes to their poor viral clearance, increased corneal pathology, and reduced survival described in (**Figures 1B–E**). These results highlight a central role for the CCL25 axis in orchestrating protective local T cell immunity against HSV-1.

### 3 CCL25 deficiency reduces trigeminal ganglia CD4^+^ and CD8^+^ T cell infiltration, activation, and tissue-resident memory signatures following ocular HSV-1 infection

**Figure 3:**
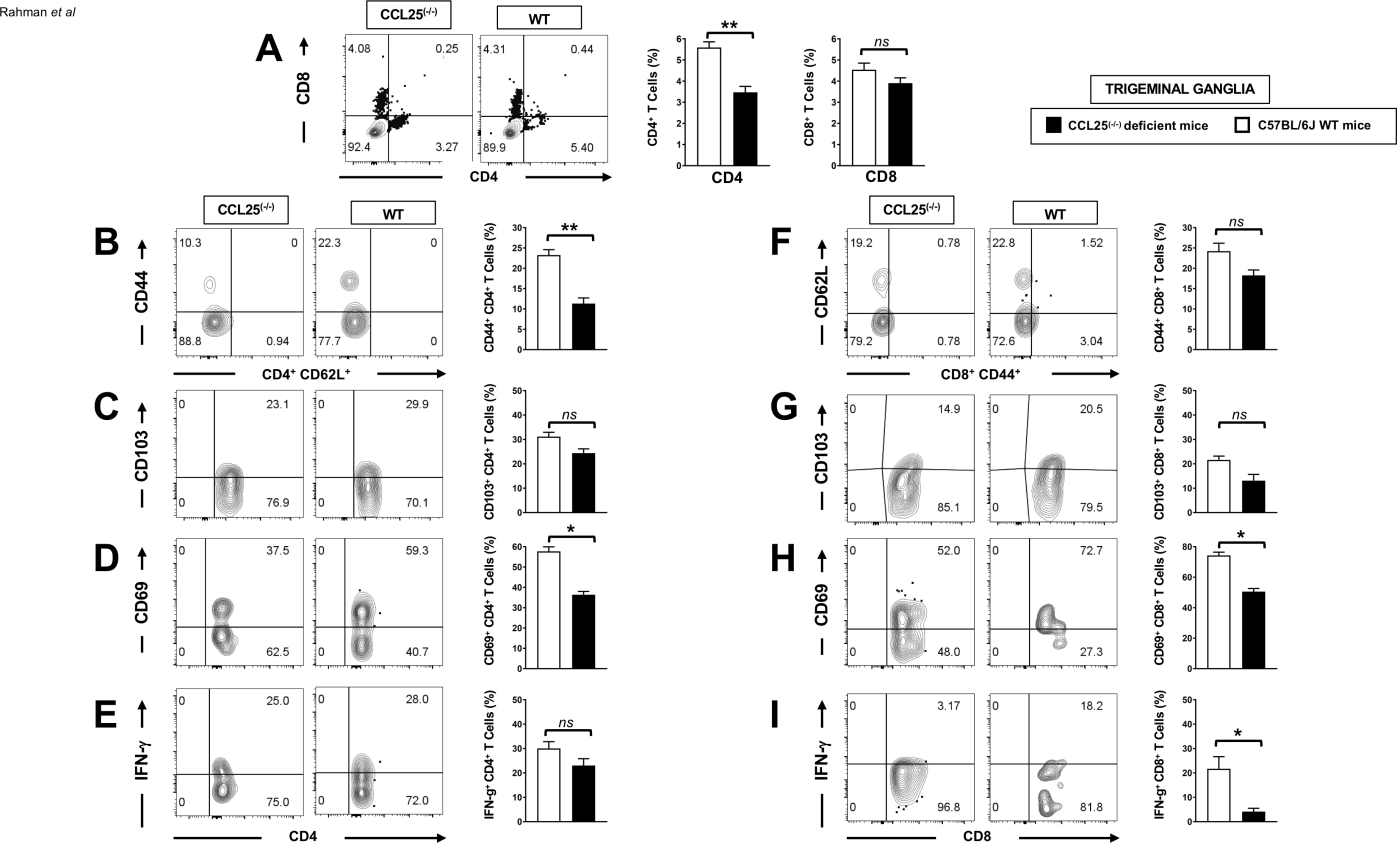
CCL25 Promotes Activated, Tissue-Resident, and Functional T Cell Accumulation in the Trigeminal Ganglion. CCL25 deficiency modifies T cell responses in the trigeminal ganglia during HSV-1 infection. **(A)** Flow cytometry revealed increased CD4^+^ and CD8^+^ T cell infiltration in CCL25^(–/–)^ TG compared to WT. **(B, E, F)** Phenotypic analysis showed a higher frequency of CD44+ effector-memory T cells in WT mice. **(C, G, D, H)** CD103+T and CD69+T cell expression were significantly higher in WT TG, CD4+, and CD8^+^ subsets. **(E, I)** Despite these changes, fewer CD4+T and CD8+T cells produced IFN-γ in CCL25^(–/–)^ deficient mice than in WT. *P ≤ 0.05, **P ≤ 0.01.

**Figure 4:**
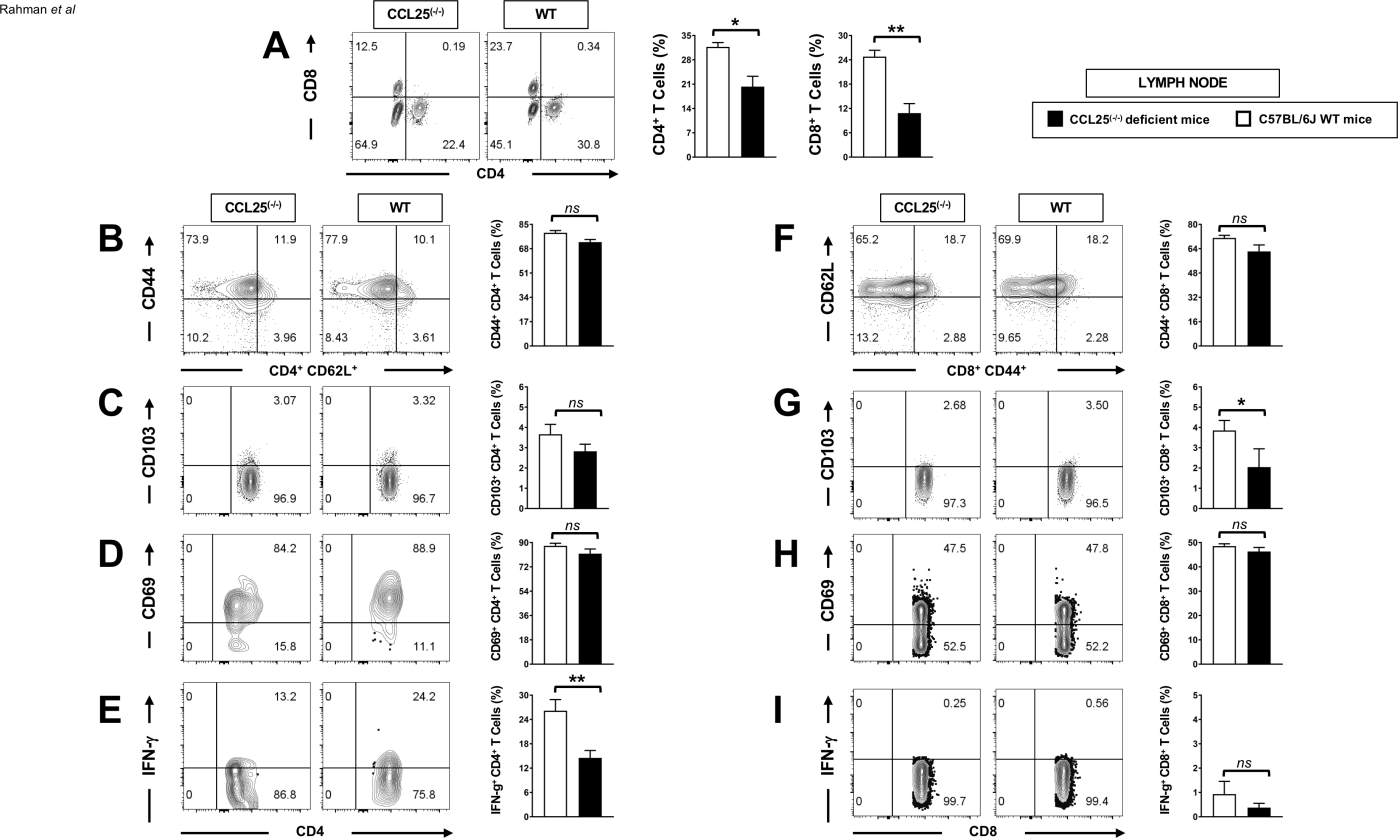
Phenotypic and functional characterization of CD4^+^ and CD8^+^ T cells in the cervical lymph nodes. Phenotypic and functional characterization of CD4^+^ and CD8^+^ T cells in the cervical lymph node. Single-cell suspensions from cervical lymph nodes were stained for surface markers and intracellular cytokines and then analyzed by flow cytometry. Representative contour plots show the gating strategy and the expression of activation, tissue residency, and effector function markers. First, **(A)** lymphocytes were gated and separated into CD4^+^ and CD8^+^ subsets. **(B)** Within CD4^+^ T cells, memory subsets were defined by CD44+ expression, revealing the proportions of effector memory (CD44^+^CD62L^–^). **(C, D)** CD103 expression was assessed to identify CD4^+^ tissue-resident memory (T_RM_) features, while CD69 expression further activated the status. **(E)** Intracellular IFN-γ production by CD4^+^ T cells was measured to determine the functional cytokine output. **(F)** Similarly, CD8^+^CD44^+^ T cells were analyzed to identify effector memory subsets. **(G, H)** Flow cytometric analysis of CD8^+^ T cells for expression of CD103 and CD69. Finally, IFNγ expression was examined in CD8^+^ T cells to evaluate cytotoxic cytokine production. The percentages displayed in each quadrant represent the mean values from representative mice, highlighting distinct phenotypic and functional differences between groups. *P ≤ 0.05, **P ≤ 0.01.

To determine whether CCL25 shapes antiviral T cell responses at the neuronal site of HSV-1 latency, we examined infiltrating T cells in the trigeminal ganglia (TG) of WT and CCL25^(–/–)^ deficient mice. Flow cytometric analysis revealed that WT mice TG contained more CD4^+^ T cells than CCL25 KO mice, while CD8^+^ T-cell levels showed no significant difference (**Figure 3A**). Phenotypically, transitional CD44^+^ T effector memory (T_EM_) cells were found to be expressed at a reduced frequency in CCL25^(−/−)^ mice, while the WT mice showed a higher frequency of CD44^+^ T_EM_ cells in both CD4^+^ and CD8^+^ compartments (**Figures 3B, F**). Markers of tissue residency were also enhanced: CD103 (**Figures 3C, G**), and CD69 (**Figures 3D, H**) were expressed at higher levels in WT TG, in both CD4^+^ and CD8^+^ T cells, suggesting stronger T_RM_ formation and retention. Despite this increase in infiltration and residency, functional analysis revealed reduced IFN-γ production in TG of WT mice compared with CCL25^(–/–)^ deficient mice, affecting both CD4^+^ and CD8^+^ subsets (**Figures 3E, I**). Thus, while CCL25 deficiency promotes effector-memory differentiation and T_RM_ establishment in the TG, it compromises their antiviral capacity, highlighting a dual role for CCL25 in balancing T cell persistence and function at the site of latency. While CD103 expression (**Figures 3**, **4**) and CD69 expression (**Figure 4**) trended higher in WT compared to CCL25^(–/–)^ deficient mice, these differences did not reach statistical significance. This may reflect a modest effect size of CCL25 deficiency on tissue-residency marker upregulation at the time points examined, inherent variability in tissue-resident memory T-cell establishment during acute HSV-1 infection, or insufficient statistical power to detect smaller differences in these populations. Collectively, these findings suggest that CCL25 may contribute more prominently to the initial recruitment of effector T cells than to their subsequent acquisition of tissue-residency markers.

This result highlights that CCL25 is not just a trafficking chemokine, but a regulator of the quality of antiviral T cell responses in neuronal tissue. It balances quantity versus function; too little CCL25 allows more cells to enter and differentiate, but at the cost of losing optimal antiviral activity.

### 4 Loss of CCL25 diminishes T cell activation, effector differentiation, and memory formation in cervical lymph nodes following ocular HSV-1 infection

In the cervical lymph nodes, CCL25 deficiency altered both the magnitude and phenotype of antiviral T cell responses. Compared with WT controls, CCL25^(–/–)^ deficient mice showed a lower abundance of both CD4^+^ and CD8^+^ T cells, indicating enhanced T cell accumulation in the draining lymphoid tissue.

Phenotypic analysis showed that both CD4^+^ and CD8^+^ T cells were present at higher frequencies compared to CCL25^(–/–)^, with CD8^+^ T cells showing the most significant increase (**Figure 4A****).** Phenotypic analysis further revealed a reduced frequency of CD44+ T cells in CCL25^(–/–)^ deficient mice. In contrast, WT mice had a relatively higher proportion of CD44^+^ T_EM_ cells in both CD4^+^ and CD8^+^ compartments (**Figures 4B, F**), indicating a more substantial shift toward effector differentiation. In the cervical lymph node, the markers of tissue residency also highlighted a genotype-dependent difference. Among CD4^+^ T cell subsets, CD103 and CD69 expression were relatively higher in WT mice than in CCL25^(–/–)^ deficient mice (**Figures 4C, D**). The CD103 and CD69 expression was higher in WT mice compared to CCL25^(–/–)^ deficient mice, particularly among CD8^+^ subsets, suggesting enhanced generation or retention of corneal T_RM_ cells (**Figures 4G, H****).** Despite these increases in effector-memory and T_RM_ populations, functional analysis showed that fewer CCL25^(–/–)^ T cells produced IFN-γ than WT in both CD4^+^ and CD8^+^ compartments (**Figures 4E, I**).

### 5 Gut-derived CCL25 regulates mucosal chemokine networks during ocular HSV-1 infection: CCL28 and CXCL14 expression are CCL25-dependent

**Figure 5:**
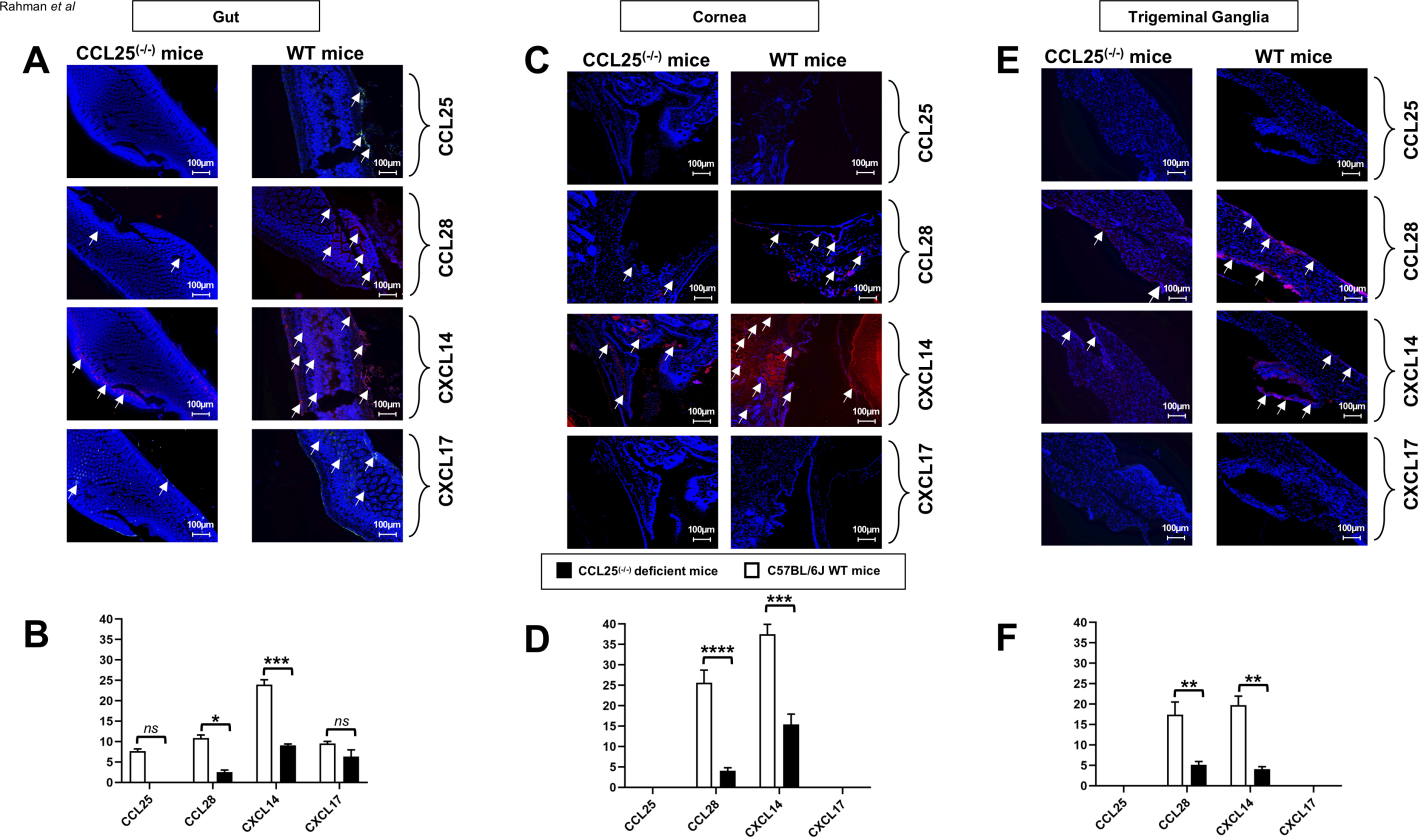
CCL25 deficiency alters chemokine expression in the small intestine and trigeminal ganglia after HSV-1 infection. CCL25 deficiency alters chemokine expression in the small intestine and trigeminal ganglia after HSV-1 infection. **(A)** Immunofluorescence staining of small intestine sections. WT mice showed strong signals for CCL25, CCL28, CXCL14, and CXCL17 (arrowheads), while CCL25^(–/–)^ deficient mice lacked CCL25 and had weaker expression of the other chemokines. **(B)** Quantification of minor intestine staining confirmed significantly higher levels of CCL28, CXCL14, andCXCL17 in WT mice compared to CCL25^(–/–)^ deficient mice, indicating that CCL25 helps regulate other mucosal chemokines. **(C, E)** Immunofluorescence of cornea and trigeminal ganglia (TG). WT mice expressed higher levels of CCL28, CXCL14, and CXCL17 than CCL25^(–/–)^ deficient mice, whereas CCL25 itself was not detected in TG, consistent with its restricted expression pattern. **(D, F)** Quantification of cornea and TG staining showed the same trend, with WT mice displaying markedly higher CCL28 and CXCL14 compared to CCL25^(–/–)^ deficient mice. *P ≤ 0.05, **P ≤ 0.01, ***P ≤ 0.001, ****P ≤ 0.0001.

We next determined the effect of CCL25 deficiency on HSV-1-mediated herpes infection and disease. For this purpose, we generated CCL25^(−/−)^ mice and compared their infection levels and the severity of Herpes-like symptoms with those in wild-type C57BL/6 mice. Before the infection with the HSV-1 McKrae variant, we performed genotyping on CCL25^-/-^ mice to confirm the absence of CCL25 expression. To determine whether CCL25 deficiency affects the expression of the four major mucosal chemokines CXCL14, CXCL17, CCL25, and CCL28; small intestine (**Figures 5A, B**), cornea (**Figures 5C, D**), and TG (**Figures 5E, F**) tissues from CCL25^(−/−)^ and WT C57BL/6 mice were stained with anti-CXCL14, CXCL17, CCL25, and CCL28 antibodies.

The results showed a significant difference in the expression of mainly two mucosal chemokines, i.e., CCL28 and CXCL14, in the small intestine, TG, and cornea. Expression of CCL28 and CXCL14 was significantly higher in the WT mice in comparison to the CCL25^(−/−)^ mice. At the same time, CXCL17 specific expression was found only in the small intestine of both WT and CCL25^(−/−)^ mice.

### 6 CCL25 deficiency drives pathological inflammatory cell infiltration in the cornea and trigeminal ganglia, worsening immunopathology during ocular HSV-1 infection

**Figure 6:**
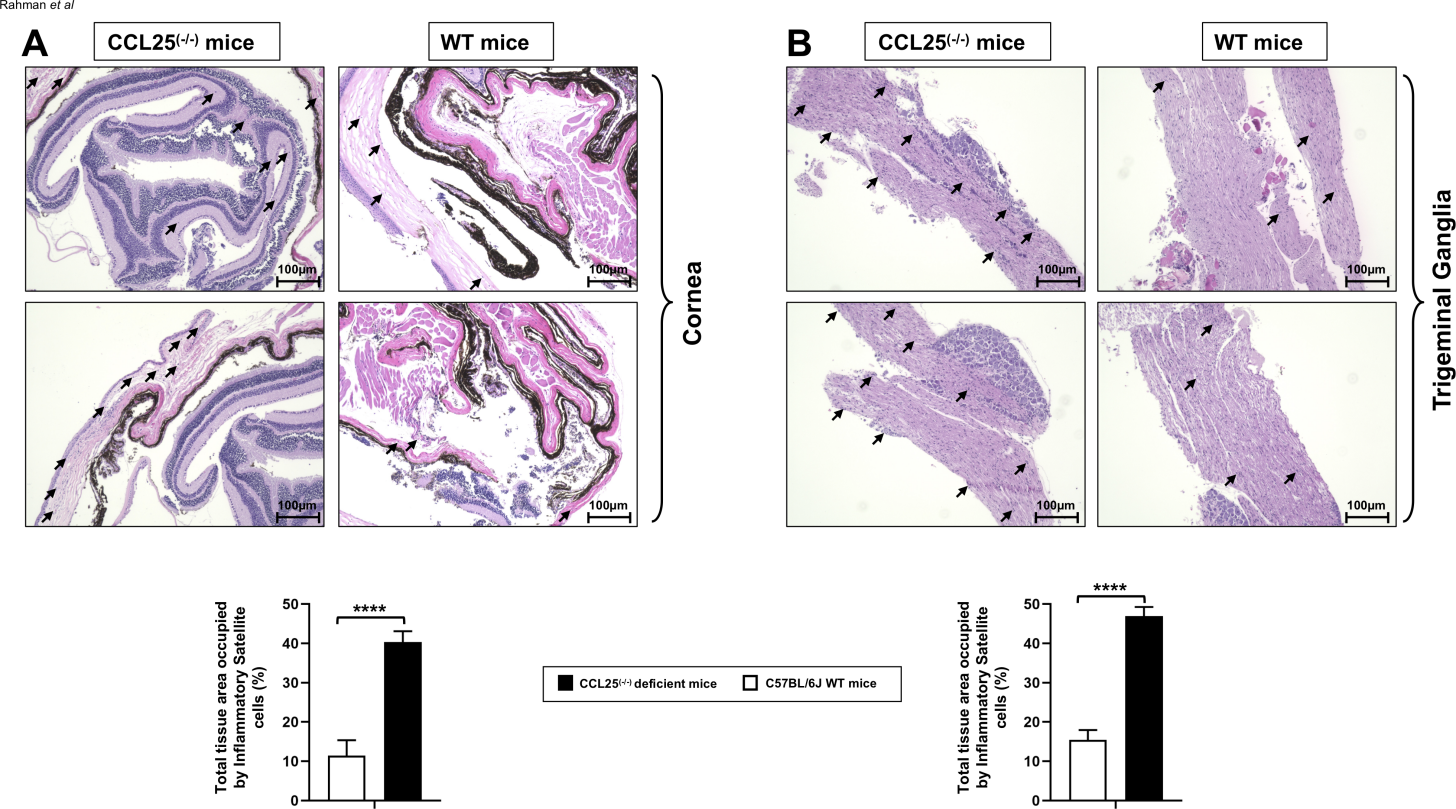
Cornea and Trigeminal Ganglia pathology following HSV-1 infection in CCL25^(–/–)^ and WT C57BL/6 mice: Representative Hematoxylin and eosin (H&E) staining images of **(A)** cornea and **(B)** TG of HSV-1 McKrae-infected mice at 4x magnification. H&E staining of corneal and trigeminal ganglia tissues reveals increased epithelial disruption and inflammation by the presence of inflammatory satellite cells (black arrows) in CCL25^(–/–)^ mice compared to WT C57BL/6 mice. The percentage of area of the total cornea and trigeminal ganglia tissue with inflammatory satellite cells is shown. Severe corneal and TG-specific pathology was observed in CCL25^(–/–)^ deficient mice. The images were taken at 4x, with a 100 μm scale.

We next determined whether CCL25 deficiency affects corneal pathology. H&E staining of cornea and TG tissue sections in HSV-1-infected CCL25^(−/−)^ mice and WT C57BL/6 mice showed a significantly higher degree of inflammation associated with severe herpes-related corneal and neuronal (TG) pathology in the CCL25^(−/−)^ mice compared to WT C57BL/6 mice (**Figures 6A, B**). A significantly higher percentage of area occupied by inflammatory satellite cells, implying a higher degree of inflammation, was observed in the cornea and TG of CCL25^(−/−)^ mice. We found a significantly reduced percentage area of inflammatory satellite cells in the cornea (**Figure 6A**) and TG (**Figure 6B**) of WT C57BL/6 mice. The images were captured at 4X resolution (**Figures 6A, B**).

## Discussion

This study supports a model in which CCL25 chemokine enhances immunity against ocular HSV-1 infection. Although CCL25 itself is not expressed in the infected eye, its abundant production in the gut (and to a lesser extent in the thymus) creates an immunological milieu that enhances the mobilization of antiviral T effector memory cells to ocular tissues (32, 33). In our experiments, WT mice (with normal CCL25) exhibited lower corneal viral loads, milder corneal pathology, and improved survival compared to CCL25^(–/–)^ deficient mice, highlighting the functional importance of this chemokine in systemic antiviral defense. The presence of more CD4^+^ and CD8^+^ effector-memory T cells in WT corneas and trigeminal ganglia correlated with faster virus clearance and less tissue damage in those tissues. These results demonstrate that even at a site where CCL25 is not produced, this chemokine can significantly influence the outcome of infection through its systemic effects. This underscores a broader immunological principle: chemokine signals generated in one tissue compartment can profoundly shape immune readiness at distant, immune-privileged sites.

In the present study, we report that CCL25 deficiency affects the expression of two major mucosal chemokines, CCL28 and CXCL14. While we fully acknowledge that the current data remains associative, definitively resolving the hierarchical relationship between CCL25 signaling and these mucosal chemokines, whether through *in vitro* CCR9 stimulation models or targeted silencing approaches, requires a dedicated mechanistic investigation beyond the scope of this foundational study. One intriguing aspect of our findings is the pronounced upregulation of CCL28 and CXCL14 in WT mice during infection. This suggests that CCL25 may act upstream of a broader chemokine cascade. In other words, CCL25 might “prime” the immune system such that upon HSV-1 infection, other chemokine pathways are more robustly activated. For instance, CCL28 (the ligand for CCR10) is known to help regulate immune responses in epithelial-rich mucosal tissues. A recent study on genital herpes found that CCL28–CCR10 interactions facilitate the mobilization of memory CD8^+^ T cells to the vaginal mucosa, thereby enhancing protection against HSV-2 (24). Our data suggest that a similar chemokine relay may operate in ocular HSV-1 infection, in which CCL25 primes systemic immunity and subsequently amplifies recruitment mediated by CCL28, CXCL14, and CXCL17. This layered response may represent an adaptive mechanism that ensures rapid effector deployment in non-mucosal tissues such as the cornea and trigeminal ganglion. The functional dissection of the CCL25/CCR9 axis and its downstream chemokine network following ocular herpes infection will therefore be rigorously addressed in our planned follow-up work and be the subject of a future report. Our observation of increased CCL28 expression in WT mice aligns with this concept and suggests that a similar mechanism may operate in ocular immunity, whereby mucosal chemokines help attract effector T cells to the site of viral infection. Likewise, CXCL14 and CXCL17 are homeostatic chemokines associated with mucosal surfaces and myeloid cell recruitment; their elevation in WT tissues could contribute to a more effective innate immune environment that supports T cell function during HSV-1 infection (34, 35).

**Figure 7:**
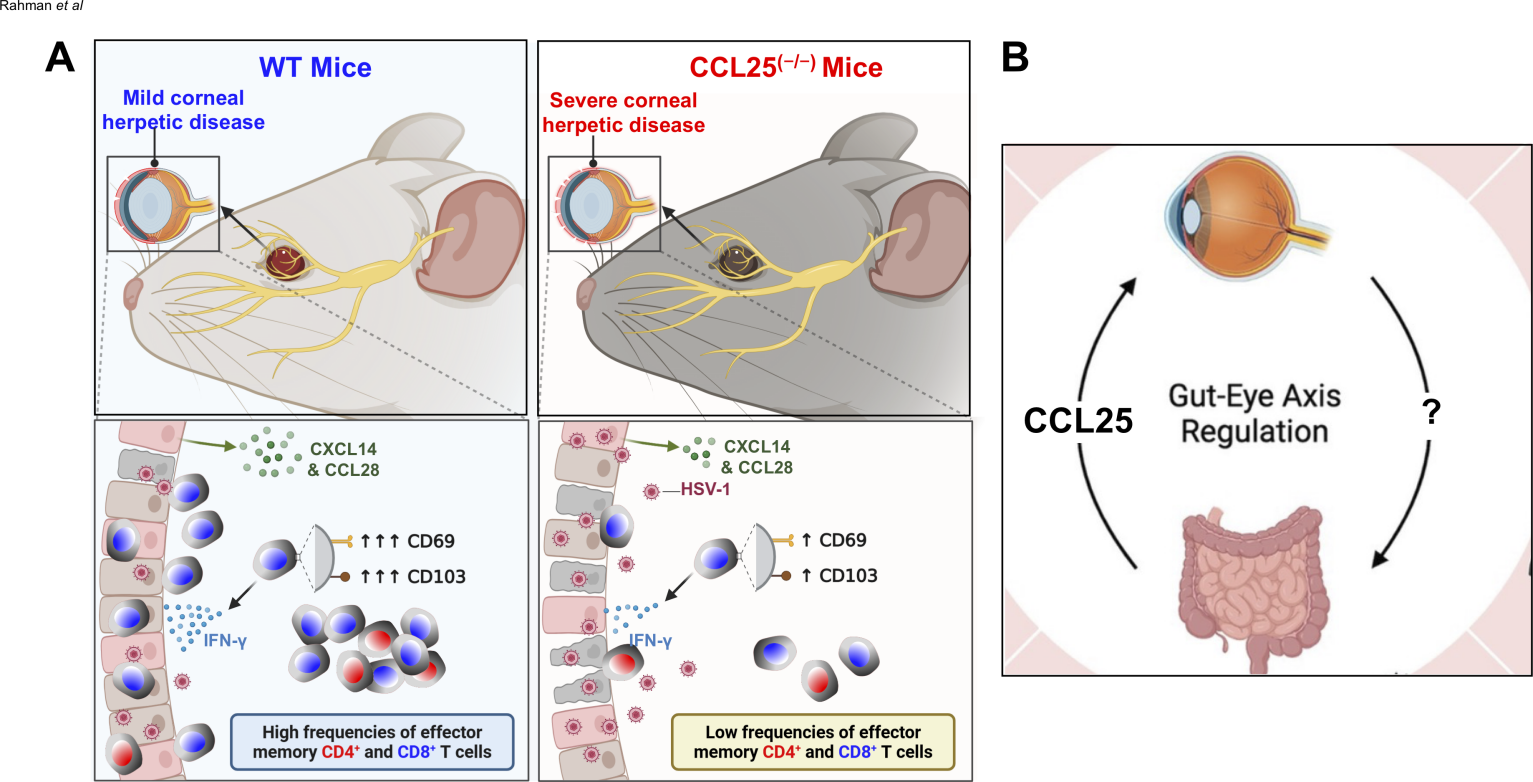
Illustration of the working model by which the CCL25/CCCR9 chemokine axis promotes protective tissue-resident memory CD4^+^ T and CD8^+^ T cells in the eye. **(A)** Mild ocular herpetic disease developed by wild-type (WT) C57BL/6J mice following ocular inoculation with HSV-1 (McKrae strain, *top left*) compared to severe cornea herpetic disease developed by age-matched CCL25^(−/−)^ deficient mice (*top right*). Compared to wild-type (WT) C57BL/6J mice (*bottom left*), the infected cornea of CCL25^(–/–)^ deficient mice exhibited reduced frequencies of antiviral effector memory CD44^+^CD4^+^ and CD44^+^CD8^+^ T cells, fewer activated, interferon-γ-producing CD103^+^CD69^+^CD4^+^ and CD103^+^CD69^+^CD8^+^ T cells, and high levels of CXCL14 and CCL28 mucosal chemokines (*Bottom right).* **(B)** The image depicts the “Gut-Eye Axis Regulation,” illustrating a connection between the gut (represented by the intestines) and the eye. This axis refers to the bidirectional communication and influence between the gut microbiome and ocular health. Mucosal CCL25 chemokines produced in the gut influence ocular herpesvirus infection and immunity. Whether chemokines or other factors produced in the eye affect the gut infection and immunity remains to be determined. Understanding the role of mucosal chemokines in the gut-eye axis opens avenues for therapeutic interventions targeting gut-derived chemokines to treat eye conditions. Figure created with BioRender.com.

The present study was designed as a foundational report that supports the concept that gut-derived chemokines shape antiviral ocular immunity. While our findings support an association between CCL25 deficiency and impaired antiviral immunity during ocular HSV-1 infection, we acknowledge that the precise mechanistic basis of CCL25/CCR9-mediated protection remains to be fully elucidated. Future studies will directly address this by employing CCR9 blockade, CCL25 signaling rescue approaches, and adoptive transfer of CCR9-expressing T cells between wild-type and CCL25-deficient mice. These experiments will determine whether the observed protective phenotype is specifically attributable to the CCL25/CCR9 signaling axis, rather than reflecting broader developmental or systemic immune consequences of CCL25 deficiency. The mechanism by which CCL25 exerts these systemic effects would be consistent with a potential gut-eye immune connection through a role of the CCL25/CCR9 axis in T cell trafficking and tissue imprinting. Within gut-associated lymphoid tissues, CCL25 helps retain and attract CCR9^+^ T cells, and intestinal dendritic cells can produce retinoic acid to imprint T cells with gut-homing markers like CCR9 and α4β7 (22, 36–38). In WT mice, this imprinting may result in a larger pool of effector T cells that reside in or circulate through mucosal sites and are poised to respond to HSV-1. When an infection occurs in the eye (which lacks CCL25), the presence of infection-induced chemokines, such as CCL28 and CXCL17, can recruit these effector T cells from the circulation to the eye (25, 39). In CCL25^(–/–)^ deficient mice, however, fewer T cells exhibit gut-tropic (mucosal-conditioned) phenotypes, resulting in a weaker systemic T cell response and reduced trafficking of effectors to the eye. Additionally, CCL25 produced in the thymus might influence the development or egress of specific T cell subsets, potentially affecting the repertoire of memory T cells available to combat HSV-1 in peripheral tissues (40). The observed trends toward higher CD103 and CD69 expression in WT compared to CCL25^(–/–)^ deficient mice, while not statistically significant, warrant further consideration. The absence of significance may be attributed to the relatively modest effect of CCL25 deficiency on tissue-residency marker upregulation at the examined time points, the inherent biological variability associated with tissue-resident memory T-cell establishment during acute HSV-1 infection, or limited statistical power in detecting subtle differences within these populations. Importantly, these findings suggest that CCL25 plays a functionally distinct role in the early recruitment of effector T cells versus their later differentiation into tissue-resident memory cells, as illustrated in the working model in **Figure 7**. This distinction is important, as the mechanisms governing T-cell recruitment and tissue-residency programming are not necessarily coupled. However, one limitation of the current study is the exclusion of baseline (pre-infection) levels of immune cell populations following HSV-1 infection. Future mechanistic studies will directly examine this question to more precisely define the contribution of CCL25 to each stage of the tissue-resident memory T-cell response. Moreover, the precise step in the effector T-cell cycle governed by CCL25/CCR9 signaling, whether trafficking, tissue retention, local activation, and/or survival of T cells are affected, remains to be fully resolved (**Figures 7A, B**). Future follow-up mechanistic studies will address these questions directly, employing intravital imaging, proliferation and survival assays, and time-course analyses of CCR9-expressing CD4^+^ and CD8^+^ T cell infiltrations in the cornea and TG, which will form the basis of dedicated future reports.

These findings have important translational implications. Enhancing CCL25 signaling, or the downstream chemokine network it influences, could be a strategy to boost systemic antiviral immunity (33). For example, mucosal vaccination approaches might be designed to elevate CCL25 levels or to imprint vaccine-induced T cells with gut-homing and effector-memory properties. An ideal HSV-1 vaccine could include an adjuvant that induces CCL25 expression (or mimics its action), enabling vaccinated individuals to develop circulating T effector memory cells that can rapidly access the eye and trigeminal ganglion in the event of infection. Conversely, therapeutic administration of CCL25 or CCR9 agonists might be explored to enhance T cell recruitment during acute ocular herpes outbreaks. Any such intervention would need to be balanced against potential risks, since excessive chemokine signaling can also drive immunopathology. Future studies will be required to determine whether pharmacologic modulation of CCL25 pathways can achieve protective effects without exacerbating tissue inflammation.

Given the global burden of HSV-1-related blindness, an estimated 1.5 million new ocular HSV cases and 40,000 cases of severe vision loss each year (1, 33, 41), new strategies to improve protective immunity are greatly needed. No effective HSV-1 vaccine is currently available, and recurrent herpetic disease remains a significant clinical challenge (42). Our work uncovers a previously unappreciated link between gut mucosal immunity and outcomes of ocular infection. It underscores that enhancing immune responses in one tissue compartment (e.g., the gut) can have far-reaching benefits at an immune-privileged site, such as the cornea.

In summary, CCL25 promotes the systemic mobilization of antiviral effector memory T cells, enabling their efficient recruitment to the eye and the trigeminal ganglion during HSV-1 infection, thereby limiting herpetic disease. This mechanism highlights the interconnectedness of mucosal and systemic immunity, offering a new perspective on harnessing chemokines for immune interventions against viral infections that threaten vision. Taken together, these results not only support the concept of a gut-eye axis in HSV−1 infection and immunity but also open avenues for translational research into chemokine-based vaccines and therapeutics that could fundamentally shift the way we prevent and treat ocular herpes.

## Data Availability

The raw data supporting the conclusions of this article will be made available by the authors, without undue reservation.
