# Supplementary material for: CCL25 chemokine promotes antiviral tissue resident CD4+ and CD8+ effector memory TRM cells associated with a reduction of ocular herpes infection and disease: a potential gut–eye axis in herpes immunity
Source: Front Immunol. 2026 Jul 17;17:1872553. doi: 10.3389/fimmu.2026.1872553 (PMC13425080; doi:10.3389/fimmu.2026.1872553)
Supplement: Supplementary file 1 [file DataSheet1.pdf]

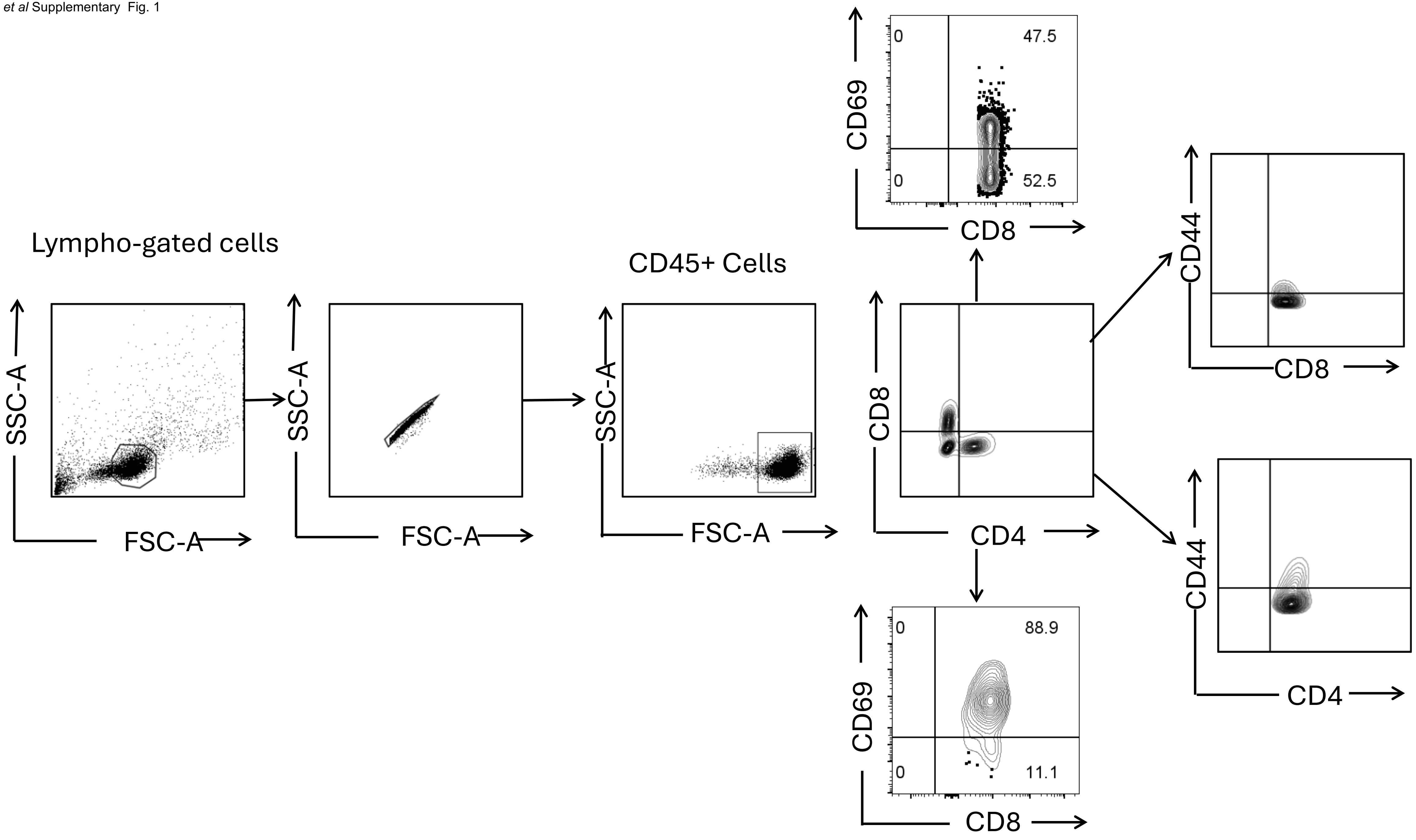

Supplementary Figure 1. Gating Strategy: Example of a gating strategy applied when analyzing the flow cytometry data presented in this study.
